# Persistent Varicella Transmission in a High-Coverage Single-Dose Vaccination Program: Costa Rica, 2015–2024

**DOI:** 10.3390/vaccines14100909

**Published:** 2026-10-19

**Authors:** Oscar Hidalgo-Mora, Helena Brenes-Chacón, Sebastián Ospina-Henao, Alejandra Soriano-Fallas, Gabriela Naranjo-Zúñiga, Gabriela Ivankovich-Escoto, María L. Ávila-Agüero

**Affiliations:** 1 Department of Pediatrics, Hospital Nacional de Niños “Dr. Carlos Sáenz Herrera”, Caja Costarricense de Seguro Social (CCSS), San Jose 10101, Costa Rica; ofhidalgo@ccss.sa.cr (O.H.-M.); givankovich@ccss.sa.cr (G.I.-E.); 2 Department of Pediatric Infectious Diseases, Hospital Nacional de Niños “Dr. Carlos Sáenz Herrera”, Caja Costarricense de Seguro Social (CCSS), San Jose 10101, Costa Rica; helenabrenes@gmail.com (H.B.-C.); asoriano@ccss.sa.cr (A.S.-F.); 3 Faculty of Medicine, Universidad de Ciencias Médicas (UCIMED), San Jose 10101, Costa Rica; dr.ospinahenao@gmail.com (S.O.-H.); mgnaranjoz@ccss.sa.cr (G.N.-Z.); 4 Center for Infectious Disease Modeling and Analysis, Yale University School of Public Health, New Haven, CT 06520, USA

**Keywords:** varicella, vaccination, prevention, Costa Rica, Latin America

## Abstract

**Background:** Costa Rica introduced universal one-dose varicella vaccination in 2007 using a one-dose schedule administered at 15 months of age through the Costa Rican Social Security Fund (CCSS). A previous national study described epidemiological changes through 2014 by comparing pre- and post-vaccination periods; however, contemporary data on subsequent patterns and residual disease burden are limited. We characterized varicella epidemiology and healthcare burden in Costa Rica using the most recent nationally available data. **Methods:** We conducted a nationwide retrospective descriptive study using aggregated surveillance and administrative data. Outcomes included incidence of reported varicella cases (2015–2022), outpatient and emergency department visits (2019–2024), hospitalizations (2015–2024), in-hospital deaths with a recorded varicella diagnosis (2018–2020), institutional outbreaks, and first-dose vaccination coverage (2018–2024). **Results:** From 2015 to 2022, reported incidence declined by 84% in males and 83% in females, after increasing through 2019 and declining markedly during the COVID-19 pandemic. Children aged 1–9 years accounted for the largest proportion of reported cases. Among 354 hospitalizations, adults aged 20–44 years accounted for 30.5%. Hospitalizations among children aged ≤4 years decreased by approximately 56% between 2015–2018 and 2021–2024, while annual hospitalizations among adults aged 20–44 years increased from 7 in 2015 to 21 in 2024, representing a 200% increase over the study period. Three deaths occurred during hospitalizations with a varicella diagnosis, all among adults, and one institutional outbreak was identified. First-dose vaccination coverage averaged approximately 94% during 2018–2022 and remained high thereafter. **Conclusions:** Reported varicella incidence declined substantially during the study period, but circulation and healthcare burden persisted, with hospitalizations increasingly concentrated among adults. Because our analysis lacks a pre-vaccination comparator, these trends cannot be causally attributed to vaccination based on our data alone. The findings support maintaining high first-dose coverage and strengthening surveillance. Further information on individual vaccination history and population susceptibility is needed to evaluate the potential contribution of a two-dose vaccination strategy in Costa Rica.

## 1. Introduction

Varicella is a highly contagious vaccine-preventable disease caused by primary infection with varicella-zoster virus (VZV), a human alpha herpesvirus. Transmission occurs primarily through the respiratory route and direct contact with vesicular fluid, and the disease typically presents with fever and a generalized pruritic vesicular rash [1]. Although generally self-limited, complications and death may occur, including among previously healthy individuals, and primary infection in adults is associated with greater clinical severity [1,2,3]. These characteristics make surveillance and sustained population-level prevention important components of varicella control.

Universal childhood vaccination has substantially reduced varicella incidence, healthcare utilization, hospitalizations, and mortality in countries where high coverage has been maintained [4,5] and has been increasingly incorporated into national immunization programs, including across Latin America and the Caribbean [6]. A meta-analysis estimated that one vaccine dose provides approximately 81% effectiveness against varicella of any severity and 98% against moderate-to-severe disease, whereas two doses provide greater protection against disease of any severity [7]. The World Health Organization considers one dose sufficient when the principal objective is reducing severe disease and mortality, while a two-dose schedule is preferable when the programmatic objective includes further reduction in cases and outbreaks [4].

Costa Rica introduced universal childhood varicella vaccination in 2007 as a one-dose schedule administered routinely at 15 months of age through the publicly funded National Immunization Program [8]. Before vaccine introduction, an estimated 600–800 cases per 100,000 population occurred annually [8]. A previous national evaluation comparing the pre-vaccination and early post-vaccination periods documented a 73.8% reduction in reported cases and an 85.9% reduction in hospitalizations in the general population, together with substantial declines among children aged <5 years and in complicated pediatric cases [8]. Vaccination coverage increased from 76% in 2008 to approximately 95% in 2015 [8].

Several Latin American and Caribbean countries have incorporated universal varicella vaccination using one- or two-dose schedules. Similar reductions in varicella incidence and hospitalizations following vaccine introduction have been reported across the region [9,10,11,12]. Nevertheless, regional experience has been heterogeneous because of differences in vaccination schedules, coverage, surveillance systems, and the availability and completeness of epidemiological data. Consequently, country-specific evaluations remain necessary to characterize national epidemiological patterns and inform vaccination policies.

Following the period evaluated in the previous Costa Rican study, an integrated national assessment of varicella epidemiology and healthcare burden during the subsequent decade has been lacking. Updated information on temporal trends, age distribution, geographic variation, healthcare utilization, hospitalizations, deaths, outbreaks, and vaccination coverage is needed to identify the residual burden of disease and inform future prevention strategies. Therefore, this study aimed to describe contemporary epidemiological trends and patterns in healthcare utilization associated with varicella in Costa Rica using nationally available surveillance and administrative data for 2015–2024.

## 2. Materials and Methods

### 2.1. Study Design, Setting, and Data Sources

We conducted a nationwide retrospective descriptive epidemiological study to characterize temporal, demographic, and geographic patterns of varicella in Costa Rica from 1 January 2015 to 31 December 2024. Costa Rica maintains mandatory passive surveillance for varicella infection. In routine surveillance, diagnosis is primarily clinical, and laboratory confirmation is not systematically required. The unit of analysis consisted of aggregated population-level data.

Data were obtained from two national sources. National surveillance data from the Ministry of Health were used exclusively to estimate annual varicella incidence and included notified cases from 2015 to 2022, corresponding to the most recent years for which complete incidence data were available. All other study outcomes were obtained from administrative and institutional data from the Costa Rican Social Security Fund (Caja Costarricense de Seguro Social [CCSS]), which provides healthcare services to most of the population. CCSS data included hospital discharges with varicella as a principal or secondary diagnosis (2015–2024), in-hospital deaths with a recorded varicella diagnosis (2018–2020), outpatient consultations due to varicella (2019–2024), emergency department (ED) visits due to varicella (2019–2024), institutional outbreaks (2015–2024), and first-dose varicella vaccination coverage obtained from official reports of the CCSS National Immunization Program (2015–2024).

Data were aggregated and analyzed by age group, sex, province of residence, and reporting facility, as applicable to each dataset. Complete data were not available for all outcomes and variables across the entire study period because the availability, completeness, and continuity of routinely collected surveillance and administrative data varied across data sources and years. Analyses were therefore restricted to the periods for which data for each outcome were available.

### 2.2. Case Definition, Variables and Outcomes

Varicella cases in administrative datasets were identified using ICD-10 diagnostic codes B01.0–B01.9 recorded as principal or secondary diagnoses. Surveillance incidence data were based on clinically diagnosed cases reported through the national surveillance system. Laboratory confirmation status was not consistently available and was therefore not used as a case inclusion criterion.

Annual incidence rates were calculated using mid-year population estimates obtained from the National Institute of Statistics and Census of Costa Rica (INEC) and expressed per 100,000 population. Age was categorized as follows: infants <1 year, 1–4 years, 5–9 years, 10–14 years, 15–19 years, 20–44 years, 45–64 years, and ≥65 years.

Primary outcomes included annual varicella incidence rates, hospitalizations, outpatient visits, emergency department visits, in-hospital deaths, reported institutional outbreaks, and first-dose vaccination coverage. Vaccination coverage was obtained from official reports of the national immunization program and expressed as the annual percentage of the target birth cohort vaccinated.

In-hospital deaths were identified when a varicella ICD-10 code (B01.0–B01.9) was recorded as a principal or secondary diagnosis. The cause of death was not independently verified through individual medical record review. Institutional outbreaks were defined according to the surveillance criteria applied by the CCSS and were analyzed as reported in institutional surveillance records; no independent outbreak classification was performed by the investigators.

First-dose varicella vaccination coverage was defined as the annual percentage of the target childhood population reported as having received the first dose of varicella vaccine, as reported by the CCSS National Immunization Program. Coverage therefore represents vaccination of the program’s target population rather than the proportion of the entire Costa Rican population vaccinated against varicella. Coverage estimates were not independently calculated by the investigators; reported values were used to describe temporal and geographic trends.

### 2.3. Statistical Analysis and Data Quality

Descriptive analyses were conducted using aggregated anonymized datasets. Analyses were descriptive and were not designed to estimate individual-level associations or risk factors for disease or hospitalization. Incidence rates were calculated per 100,000 population. Counts and proportions were used to summarize hospitalizations, outpatient visits, ED consultations, deaths, and outbreaks. Outpatient and ED utilization was reported as absolute numbers of healthcare encounters rather than population-based rates. For age-specific distributions, proportions were calculated using the total number of encounters included in the corresponding analysis as the denominator. Healthcare encounters did not necessarily represent unique patients. Temporal, demographic, and geographic patterns were assessed descriptively across the available years, with analyses stratified, as applicable, by age group, sex, and province.

Percentage change between the first and last available year for each outcome was calculated to quantify relative variation over time, using the following formula:

Percentage change (%) = [(Final value − Initial value)/Initial value] × 100

To assess changes in varicella burden between the 2015–2018 and 2021–2024 periods, outcomes were compared descriptively using aggregated counts, proportions, and percentage changes, as applicable according to data availability. No formal statistical hypothesis testing was performed for these comparisons.

Data management, descriptive statistical analyses, and generation of summary tables were performed using Microsoft Excel for Microsoft 365 (Microsoft Corporation, Redmond, WA, USA).

Data quality assessment included cross-verification of aggregated case counts between surveillance and administrative datasets when overlapping years were available, identification of missing values in key variables, consistency checks of age-specific distributions across years to detect anomalous reporting patterns, and assessment of internal consistency between total hospitalization counts and age-stratified counts. Vaccination coverage estimates exceeding 100% were examined in relation to denominator sources and reporting practices. No imputation procedures were applied; analyses were conducted using available data.

### 2.4. Ethical Considerations

The study was approved by the Scientific Ethics Committee of the National Children’s Hospital “Dr. Carlos Sáenz Herrera”. All datasets were anonymized prior to analysis. No individual-level identifiers were accessed, and no direct review of clinical charts was performed.

## 3. Results

### 3.1. National Incidence and Geographic Distribution

**Figure 1:**
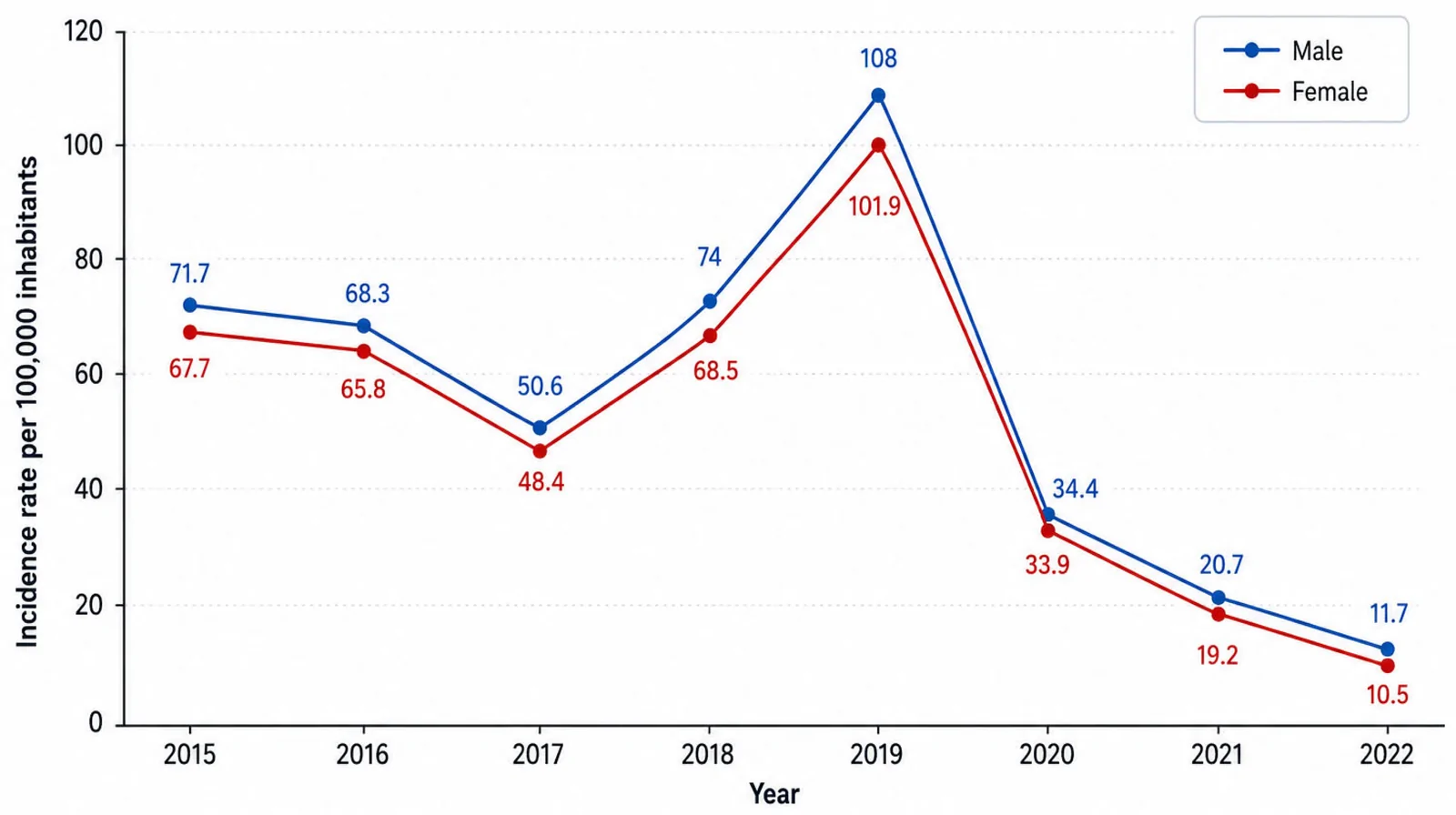
Annual varicella incidence per 100,000 population by sex in Costa Rica, 2015–2022.

Between 2015 and 2022, annual varicella incidence declined markedly in both sexes, with an overall reduction of approximately 84% in males and 83% in females. Incidence increased progressively from 2015 to a peak in 2019 (108.0 per 100,000 males; 101.9 per 100,000 females), representing the highest observed rates in the study period. From 2019 to 2022, incidence decreased by nearly 90%, reaching 11.7 and 10.5 per 100,000, respectively. A marked decline was observed during 2020–2021, coinciding with the COVID-19 pandemic. Incidence remained substantially below pre-pandemic levels in 2022 (Figure 1). Cumulatively, males accounted for a slightly higher overall burden, although sex-specific rates were comparable across years.

Geographic variation in reported varicella incidence was observed throughout the study period, with coastal and peripheral regions generally showing higher reported incidence than central regions. At the aggregate level, no consistent correspondence between regional vaccination coverage and reported incidence was observed.

### 3.2. Age-Specific Distribution

Age-stratified analyses showed that the reported disease burden was concentrated in younger populations. Infants < 1 year and children aged 1–9 years accounted for the largest proportion of reported cases. Incidence declined progressively with increasing age, reaching the lowest levels among adults aged ≥65 years. Overall, 52% of reported cases occurred in males. These findings indicate that reported varicella cases remained concentrated in younger age groups during the study period.

### 3.3. Healthcare Services Utilization

**Figure 2:**
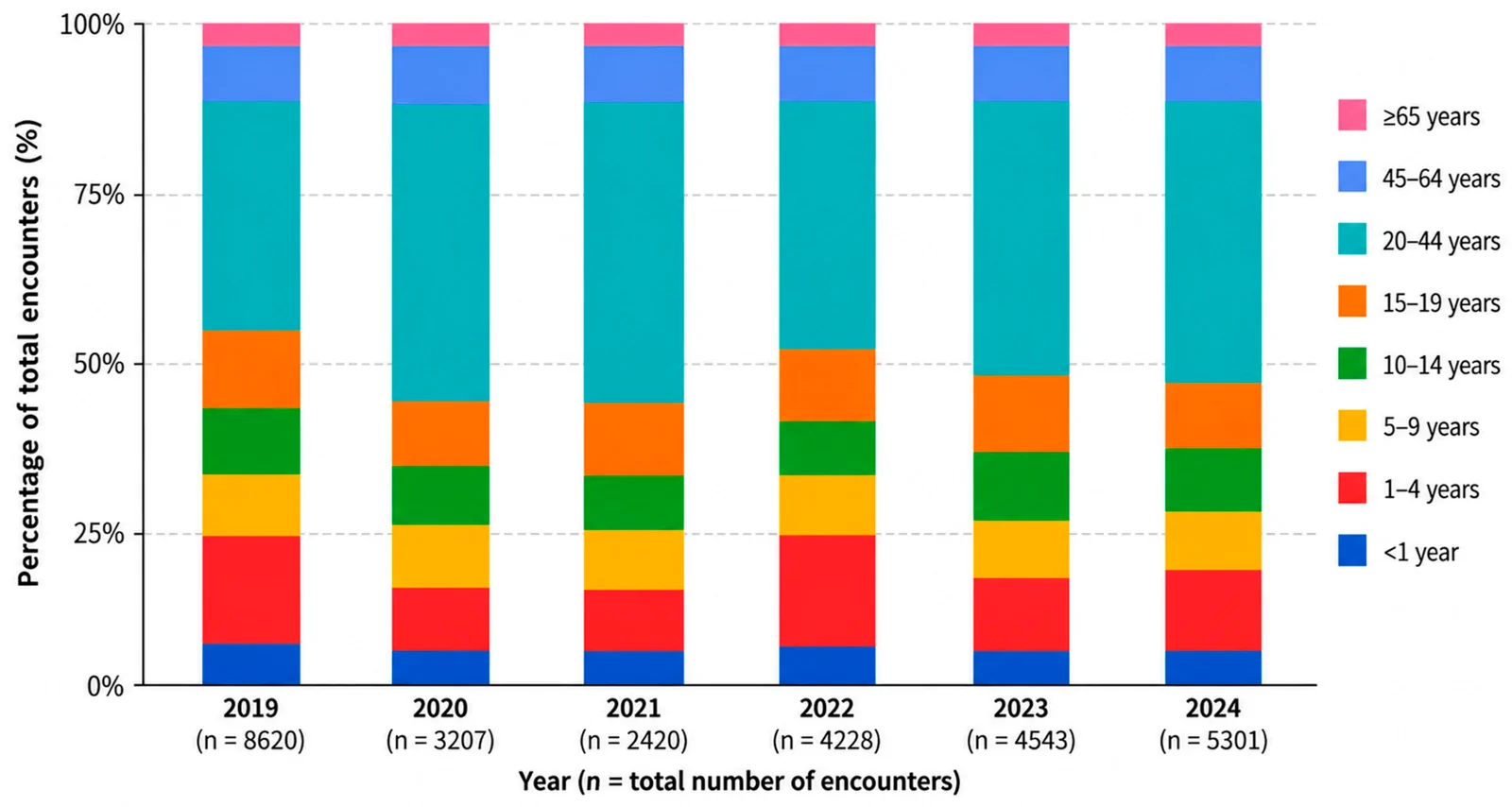
Varicella-related Healthcare Services Utilization by Age Group in Costa Rica, 2019–2024. Data represent the annual age distribution of combined outpatient and emergency department encounters. For each year, percentages were calculated using the total number of varicella-related healthcare encounters recorded in that year as the denominator. A total of 28,319 encounters were recorded during 2019–2024, including 8715 outpatient visits and 19,604 emergency department visits.

Between 2019 and 2024, 28,319 varicella-related healthcare encounters were recorded, including 8715 outpatient visits and 19,604 emergency department (ED) visits. These values represent absolute numbers of recorded healthcare encounters rather than population-based rates and may include multiple encounters by the same patient. For service-specific age distributions, the denominators were the total numbers of outpatient visits (*n* = 8715) and ED visits (*n* = 19,604), respectively. Adults aged 20–44 years accounted for the largest proportion of encounters in both settings, representing 38.8% of outpatient visits and 36.6% of ED visits. Children aged 1–4 years accounted for 15.1% and 17.6% of outpatient and ED visits, respectively (Figure 2). Healthcare utilization declined markedly during 2020–2021, particularly in ED visits, which decreased by more than 60% between 2019 and 2021, followed by a partial recovery through 2024. Despite these temporal changes, adults aged 20–44 years remained the predominant age group in both outpatient and ED settings.

### 3.4. Hospitalization

A total of 354 varicella-related hospitalizations were recorded between 2015 and 2024 (mean, 35.4 per year). Annual hospitalizations declined from 47 cases in 2018 to 15 in 2021 (68% reduction), reaching their lowest level during the COVID-19 pandemic, followed by an increase to 40 cases annually in both 2023 and 2024.

**Figure 3:**
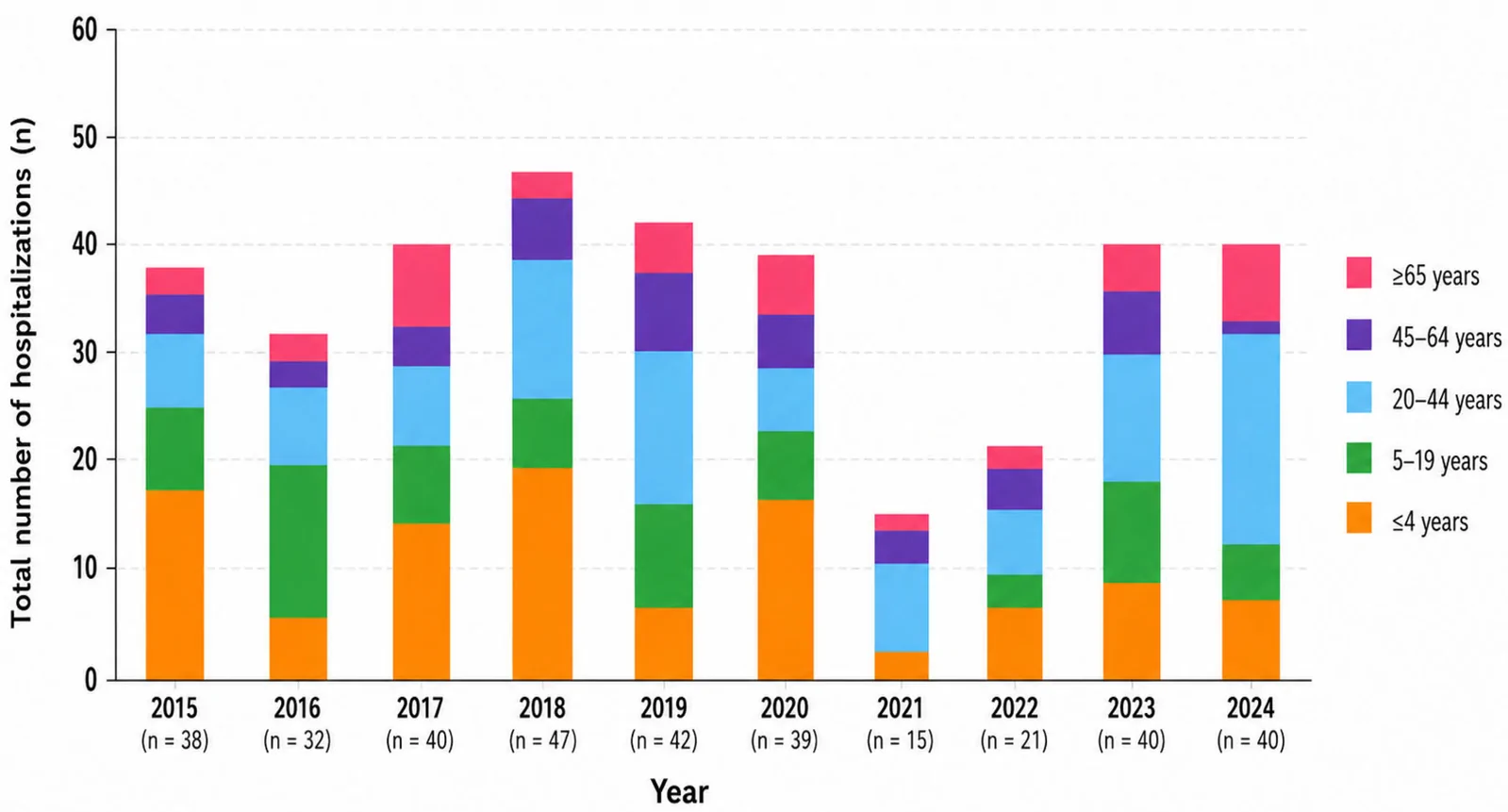
Annual hospitalizations with a varicella diagnosis by age group in Costa Rica, 2015–2024.

Adults aged 20–44 years accounted for 30.5% of total hospitalizations, becoming the leading hospitalized age group by the end of the study period. Comparing the 2015–2018 and 2021–2024 periods, hospitalizations among children aged ≤4 years decreased from 53 to 23 (approximately 56%), whereas hospitalizations among adults aged 20–44 years increased from 37 to 50 (approximately 35%). Consistent with this increasing adult burden, annual hospitalizations among adults aged 20–44 years increased from 7 cases in 2015 to 21 cases in 2024, representing a 200% increase over the study period (Figure 3).

Three deaths occurred during hospitalizations with a varicella diagnosis between 2018 and 2020, all among adults. No such deaths were identified among pediatric patients in the available dataset.

### 3.5. Outbreaks

A single institutional outbreak was identified in the available data during the study period. The outbreak occurred in 2023 in a correctional facility and involved 25 adult cases, with no reported deaths. Individual vaccination history and history of previous varicella infection were not available in the published outbreak data. This event represents the only institutional outbreak captured in the surveillance and administrative sources available for this study and should not be interpreted as the only varicella outbreak that occurred in Costa Rica during the study period. Community-, school-, and other non-institutional outbreaks could not be systematically identified from the available data.

### 3.6. Vaccination Coverage

**Figure 4:**
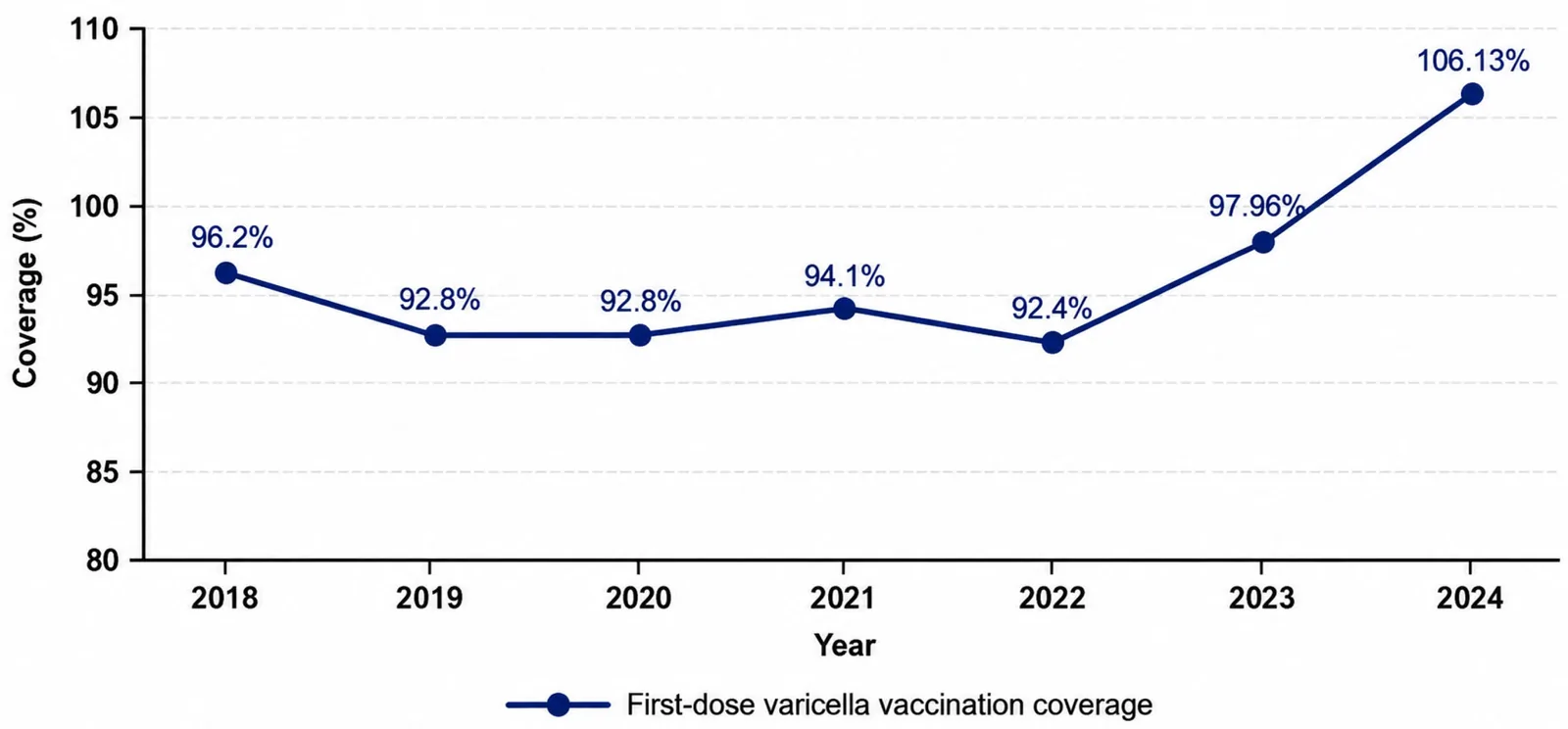
Annual first-dose varicella vaccination coverage in Costa Rica, 2018–2024.

First-dose varicella vaccination coverage showed year-to-year fluctuations during the study period. During 2018–2022, annual coverage ranged from 92.4% to 96.2%, with an average of approximately 93.7%. Reported national coverage subsequently reached approximately 98.0% in 2023 and 106.1% in 2024 (Figure 4).

Regional differences were observed in recent years, with an approximate 10-percentage-point range between the lowest and highest coverage areas. Administrative vaccination coverage values exceeding 100% can occur when the numerator used to estimate coverage and the estimated target population used as the denominator do not correspond perfectly to the same underlying population.

During the years for which both measures were available (2018–2022), short-term annual fluctuations in first-dose vaccination coverage did not show a direct temporal correspondence with reported incidence trends.

## 4. Discussion

This study provides a comprehensive description of varicella epidemiology in Costa Rica from 2015 to 2024. Substantial reductions in reported incidence and several measures of healthcare burden were observed, while reported varicella cases and healthcare utilization persisted, with hospitalizations increasingly concentrated among adults. These findings extend the epidemiological description provided by an earlier national analysis [8] and can be interpreted in the context of reports from other LAC countries that have adopted universal varicella vaccination [11,12]. Because the present analysis begins in 2015, does not include a pre-vaccination comparator, and encompasses the COVID-19 pandemic, the observed temporal trends cannot be causally attributed to the vaccination program based on our data alone.

### 4.1. Trends in Reported Varicella Incidence

Reported varicella incidence declined by more than 80% in both sexes between 2015 and 2022, although this trend was not uniform, with an increase through 2019 followed by a marked decline during the COVID-19 pandemic. This finding is consistent with the lower varicella burden previously documented in Costa Rica following vaccine introduction [8] and with reductions reported in other Latin American vaccination programs [11,12]. Geographic variation in reported incidence was also observed, with coastal and peripheral regions generally showing higher incidence than central regions. These differences should be interpreted cautiously, as geographic variation may reflect multiple factors, including differences in healthcare access, healthcare-seeking behavior, and reporting completeness, particularly in the context of passive surveillance [10].

The marked decline observed in 2020–2021 coincided with the COVID-19 pandemic. During this period, changes in social contact patterns, school closures, healthcare-seeking behavior, and case reporting may all have influenced the observed varicella trends. Reduced opportunities for transmission may have contributed to the decline, while reduced healthcare utilization and potential underreporting may have resulted in underestimation of the true disease burden. The relative contribution of these factors cannot be quantified using the available data.

### 4.2. Age Distribution and Healthcare Burden

Age-specific patterns differed according to the outcome evaluated. Reported varicella cases remained concentrated in younger age groups, particularly among infants and children aged 1–9 years, whereas adults aged 20–44 years accounted for a substantial proportion of hospitalizations. Varicella-related morbidity among Costa Rican children was documented before and during the early vaccination era, including hospitalizations and complications among immunocompetent children [13] and post-varicella acute cerebellar ataxia [14]. Together with the earlier national epidemiological analysis [8], these historical observations provide context for the lower pediatric burden observed during the present study period.

The increasing representation of adults aged 20–44 years among hospitalizations contrasts with the predominantly pediatric distribution of reported varicella cases. Although shifts in varicella burden toward older age groups have been described in other vaccination settings when susceptible individuals persist [4], our findings should not be interpreted as demonstrating such an age shift in Costa Rica. Hospitalization outcomes were analyzed using counts and proportions rather than age-specific population-based rates, and the age categories differed in width; therefore, the greater representation of adults among hospitalizations does not necessarily indicate a higher individual risk of hospitalization in this age group. Nevertheless, this pattern is clinically relevant because primary varicella infection is generally associated with greater severity and higher complication rates in adults than in children [1,4]. Given that universal childhood vaccination was introduced in 2007, many adults aged 20–44 years included in the study had already passed the routine age of vaccination when the program was implemented. However, individual vaccination histories and serological data were unavailable, preventing assessment of whether vaccination status or residual susceptibility contributed to the observed age distribution of hospitalizations.

Outpatient and emergency department data indicate that varicella continued to generate healthcare utilization during 2019–2024, with adults aged 20–44 years accounting for the largest proportion of visits in both settings. Emergency department visits should not be interpreted as a measure of severe disease, as EDs in Costa Rica may also function as rapid access points to healthcare. Reductions in varicella-related healthcare utilization following vaccine implementation have been documented in other settings [15]; however, the changes observed in our study should be interpreted descriptively rather than as a direct measure of vaccine impact.

Three deaths occurred during hospitalizations with a recorded varicella diagnosis between 2018 and 2020, all among adults, whereas no such deaths were identified among pediatric patients in the available datasets. Because varicella was recorded as either a principal or secondary diagnosis and individual medical records were not reviewed, its contribution to these deaths cannot be determined. Although previous studies have documented reductions in severe childhood varicella following universal vaccination, the present analysis cannot determine the extent to which vaccination contributed to the observed pediatric trends.

### 4.3. Vaccination Coverage and Future Vaccination Strategies

Sustaining high and equitable vaccination coverage remains an important component of varicella control [4,16] National coverage for the first dose of varicella vaccine in Costa Rica remained high during the study period, averaging approximately 93.7% during 2018–2022 and reaching approximately 98.0% in 2023 and 106.1% in 2024 [17,18]. Maintaining broad population immunity through vaccination is a fundamental principle of infectious disease prevention, although the coverage required to achieve population-level protection varies according to pathogen-specific characteristics and vaccination program factors [19,20]. Coverage values exceeding 100% should be interpreted cautiously, as administrative estimates may be affected by discrepancies between the population represented in the numerator and the estimated target population used as the denominator, potentially related to population mobility, vaccination outside the area of residence, population estimates, or other reporting and data-quality issues. The available aggregated data do not allow the contribution of these factors to be determined. Similar temporal and geographic variability in childhood vaccination coverage has been reported in Brazil [21], illustrating how national estimates may conceal subnational heterogeneity.

Evidence from studies specifically evaluating universal varicella vaccination programs provides additional context for interpreting the Costa Rican experience and for considering strategies aimed at further reducing varicella burden. Uruguay documented substantial reductions in varicella burden following implementation of universal childhood vaccination, with subsequent epidemiological assessments supporting sustained disease control [22,23,24]. Studies from Brazil have similarly demonstrated reductions in varicella-associated hospitalizations and morbidity following universal vaccination [25,26,27], while observational evidence from Panama [28] and Argentina [29] has documented substantial reductions following program implementation. In addition, modeling from Panama has suggested further potential clinical and economic benefits from increasing varicella vaccination coverage [30]. Evidence from two-dose vaccination programs and long-term vaccine studies further supports improved disease control with two doses compared with a single-dose strategy [31,32].

These international data provide a rationale for evaluating whether a two-dose strategy could offer additional population-level benefit in Costa Rica [16,17,18,31,32]; however, the present study does not establish the need for a second dose. Additional evidence on vaccination status, residual susceptibility, and breakthrough disease would be needed to inform such an evaluation. Targeted catch-up vaccination strategies for adolescents and adults without evidence of immunity, particularly in closed or other higher-risk settings, also warrant evaluation; however, our data cannot determine the size of the susceptible adult population or establish the need for a population-wide adult catch-up campaign. Maintaining high and equitable first-dose coverage and strengthening surveillance remain immediate priorities.

### 4.4. Institutional Outbreak and Implications for Closed Settings

The institutional outbreak identified in 2023 illustrates the potential for varicella transmission in closed settings and highlights the importance of coordinated outbreak preparedness and response involving healthcare and institutional authorities. However, because only institutional outbreaks captured through the available surveillance and administrative sources could be assessed, this single event should not be extrapolated to the frequency or risk of outbreaks in the general population.

### 4.5. Strengths and Limitations

The main strengths of this study are the use of national surveillance and healthcare administrative data and the inclusion of a ten-year period, providing a national overview of contemporary varicella epidemiology in Costa Rica. However, the study begins eight years after vaccine introduction and lacks a pre-vaccination comparator; therefore, it was not designed to independently estimate vaccination program impact or effectiveness. Data availability also varied by outcome: reported incidence was available for 2015–2022, hospitalizations for 2015–2024, outpatient and emergency department visits for 2019–2024, and in-hospital deaths with a recorded varicella diagnosis for 2018–2020. These differences reflect limitations in the availability, completeness, and continuity of routinely collected data, and trends outside each observation period cannot be evaluated. Incidence during 2023–2024 cannot be inferred from healthcare utilization data or directly compared with vaccination coverage estimates for those years.

Passive surveillance may underestimate varicella incidence because of underreporting and limited healthcare-seeking for mild disease. Diagnosis was primarily clinical, laboratory confirmation was not systematically required, and no case-level diagnostic validation was performed; therefore, diagnostic misclassification cannot be excluded. Administrative data may also be affected by coding or reporting inaccuracies. Outbreak ascertainment was limited to institutional events captured in the available data, and community-, school-, or other non-institutional outbreaks may therefore be underrepresented. Emergency department visits should not be interpreted as a measure of disease severity because EDs may also function as rapid access points to care.

The ecological and descriptive design precludes individual-level inference and causal attribution of the observed trends to vaccination. Healthcare utilization and hospitalization outcomes were primarily described using counts and proportions rather than age-specific population-based rates, limiting comparisons across age groups and over time. Although incidence denominators were available, the datasets did not contain the complete annual and stratum-specific information required to retrospectively calculate valid confidence intervals for all reported rates; incidence comparisons should therefore be interpreted descriptively. Individual vaccination histories, seroprevalence data, and direct measures of population susceptibility were unavailable, preventing assessment of disease burden according to vaccination status, estimation of vaccine effectiveness, and direct characterization of residual susceptibility. Similarly, the absence of linked individual-level clinical and sociodemographic information precluded multivariable assessment of factors associated with infection or hospitalization. Finally, the COVID-19 pandemic may have affected transmission, healthcare-seeking behavior, and reporting, but the relative contribution of these factors cannot be quantified.

Future studies linking surveillance and immunization records with individual clinical and sociodemographic data would enable better characterization of factors associated with varicella infection and hospitalization, residual susceptibility, and the potential contribution of future vaccination strategies.

## 5. Conclusions

This study extends the epidemiological evidence on varicella in Costa Rica by describing national trends and healthcare burden during 2015–2024, following the period evaluated in a previous national study that included pre-vaccination and early post-vaccination data. Because the present analysis begins in 2015 and does not include a pre-vaccination comparator, the observed trends cannot be causally attributed to the vaccination program based on our data alone. Nevertheless, substantial reductions in reported incidence were observed during the study period, while reported cases, healthcare utilization, and hospitalizations continued to be recorded. Reported cases remained concentrated in younger age groups, whereas adults accounted for a substantial proportion of hospitalizations. The documented institutional outbreak further illustrates the potential for varicella transmission in closed settings.

From a policy perspective, these findings support maintaining high, timely, and equitable first-dose vaccination coverage; improving the completeness, quality, and integration of vaccination and surveillance data; and strengthening preparedness and response to varicella transmission in closed institutional settings. However, the present study does not provide individual vaccination histories, seroprevalence data, or direct measures of population susceptibility and therefore cannot establish the need for a second vaccine dose. Additional evidence characterizing vaccination status and residual susceptibility is needed to evaluate targeted catch-up strategies for susceptible adolescents and adults and the potential contribution of a two-dose varicella vaccination schedule in Costa Rica. These strategies should be considered priorities for further policy evaluation rather than interventions whose necessity or effectiveness has been established by the present study.

## Data Availability

The data supporting the findings of this study were obtained from the Costa Rican Ministry of Health and the Costa Rican Social Security Fund (CCSS). Restrictions apply to the availability of these data because they contain administrative and surveillance information subject to institutional and privacy regulations. Aggregated data may be available from the corresponding author upon reasonable request and with permission from the respective institutions.

## Abbreviations

The following abbreviations are used in this manuscript:

CCSS	Costa Rican Social Security Fund
COVID-19	Coronavirus disease 2019
ED	Emergency department
ICD-10	International Classification of Diseases, 10th Revision
INEC	National Institute of Statistics and Census of Costa Rica
LAC	Latin America and the Caribbean
SLIPE	Latin American Society of Pediatric Infectious Diseases
WHO	World Health Organization
